# Self-assessed palliative care competence of healthcare professionals in a Finnish university hospital: A mixed methods study

**DOI:** 10.1177/26323524261480156

**Published:** 2026-08-25

**Authors:** Leena Vuolteenaho, Minna Hökkä, Eeva Rahko, Tarja Pölkki, Mira Rajala

**Affiliations:** 1 Research Unit of Health Sciences and Technology, MRC Oulu, 6370 University of Oulu, Oulu, Finland; 2 Research Unit of Health Sciences and Technology, University of Oulu, Oulu, Finland; 3 4170 Social and Health Care Competence Area, Kajaani University of Applied Sciences, Kajaani, Finland; 4 Department of Clinical Oncology, 60664 Oulu University Hospital, Oulu, Finland; 5 Medical Research Center Oulu, Oulu University Hospital and University of Oulu, Oulu, Finland

**Keywords:** palliative care, self-assessment, clinical competence, hospitals, university, terminal care

## Abstract

**Background:**

While the need for palliative care (PC) is growing, both national and international studies have shown insufficient PC competence among healthcare professionals. Good PC both reduces healthcare costs and promotes quality of life. It is therefore necessary to examine the current state of healthcare professionals’ level of PC competence and identify the areas in particular need of improvement.

**Objective:**

To describe the self-assessed PC competence of healthcare professionals working in a university hospital, and to study which background factors are associated with it, in order to identify areas of PC requiring improvement and further education.

**Design and methods:**

This mixed methods study with a convergent design was conducted in one hospital in Finland in 2024. The participants (n=100) were asked to rate their self-assessed PC competence using a 30-item instrument developed for the study. The data were analysed using both quantitative (descriptive statistics, comparisons between groups) and qualitative (inductive content analysis) methods.

**Results:**

Healthcare professionals’ self-assessed PC competence was “Good” or above in 18/30 items. Nurses and physicians generally rated their competence higher than did those representing other professions; competence was rated the highest in Oncology and in General Medicine and Geriatrics, respectively. Work experience in healthcare overall did not correlate with the level of competence, whereas experience in PC specifically did. In the open-ended responses, participants identified factors that promote, hinder, or otherwise impact self-assessed competence.

**Conclusion:**

PC competence was considered important, and 18/30 items were rated “Good” or “Very good”. However, the study revealed areas potentially in need of improvement and more education, both indirectly as lower scores for self-assessed competence and directly as comments provided by the participants. To provide high-quality PC, more education is required especially concerning advance care planning, guardianship, and multiprofessional cooperation. Further research is needed regarding competence among representatives of different professions.

## Plain language summary

While the need for palliative care is growing, studies have shown that healthcare professionals’ palliative care competence is insufficient. It is necessary to examine the state of healthcare professionals’ level of competence and identify the areas in need of improvement.

The objective of this study was to describe the self-assessed palliative care competence of healthcare professionals working in a university hospital, and to study which background factors are associated with it. This was done to identify areas of palliative care needing improvement and further education in order to enhance the competence of healthcare professionals, and thus to improve both the quality of healthcare and the quality of life of those receiving palliative care. The study was conducted in one hospital in Finland in 2024, with 100 participants who were asked to rate their self-assessed palliative care competence using a 30-item instrument developed for the study. The data were analysed using both quantitative and qualitative methods.

Healthcare professionals’ competence was “Good” or “Very good” in 18/30 items. Nurses and physicians generally gave their competence a higher rating than did those representing other professions; in terms of wards or units, competence was rated the highest in Oncology and in General Medicine and Geriatrics, respectively. Work experience in healthcare overall did not correlate with the level of competence, whereas experience in palliative care specifically did. In the open-ended responses, the participants identified factors that promote, hinder, or otherwise impact competence.

Competence was considered important, and 18/30 items were rated “Good” or “Very good,” but the study also revealed areas that may need improvement. More education is required especially concerning advance care planning, guardianship, and multiprofessional cooperation. Further research is needed regarding competence among representatives of different professions.

## 1. Background

Palliative care (PC) aims to improve the quality of life of patients with life-threatening illnesses, as well as that of their next of kin, by alleviating symptoms, whether physical, psychological, social, or spiritual.^1,2^ PC is not tied to the patient’s diagnosis, prognosis, or age, and may be integrated into care at any stage of illness alongside curative treatment,^3,4^ but gains more significance in the patient’s care towards the end of life.^5–7^ Care focusing on symptom relief during the last days or weeks of the patient’s life is known as end-of-life (EOL) care.^6,8–10^

The World Health Organization (WHO) considers access to PC a human right^1,11^; it is therefore imperative to be able to provide high-quality PC to those in need. The end of life is also a uniquely emotional situation for both the patient and their next of kin, as well as for the healthcare professionals involved,^12^ which further stresses the importance of competence for all parties concerned.

In the context of care, competence refers to the amalgamation of knowledge, skills, attitudes, and values required when providing care and resulting in safe, effective care.^13,14^ In this study, the criteria used for assessing PC competence specifically are based on quality recommendations for PC and EOL care^15^ and on the quality handbook for EOL care,^16^ both published by the Finnish Institute for Health and Welfare (THL). The quality recommendations were formulated as part of “The project on quality information on palliative care and end of life care”^17^ that aimed to define the data and data sources required for national quality assessment regarding PC and EOL care; the quality handbook was published alongside the quality recommendations and the final report of the project.^15–17^

Studies from various countries have revealed PC competence to be lacking and in need of improvement.^18–20^ Additionally, differences have been found in the self-assessed level of PC competence of healthcare professionals working in different healthcare settings: for example, nurses in home care feel more competent than do those working in a hospital.^21^ At present, PC education in Finland varies in scope and quality between educational institutions, both for physicians and for nurses.^22–24^ In Finland, studies on PC competence have previously been conducted with the focus on various settings^13,22,25–29^ but not, to our knowledge, on university hospitals.

According to previous studies, self-assessed PC competence may be associated with factors including education, work experience, or PC experience, although results have been partly contradictory and the need for further research has been acknowledged.^28,30,31^ Additionally, there are national and cultural differences as well as differences due to different healthcare systems in both how PC is implemented and what it is considered to entail.^10,32–35^ Therefore, even though numerous instruments for measuring PC competence have been previously developed for different contexts,^36–40^ an instrument developed for measuring specific points of interest in the current context may provide more accurate and relevant results.

Due to the ageing of the population as well as the increase in several noncommunicable diseases, the need for PC is growing,^1^ but studies have shown PC competence to require improvement, both among several professional groups in Finland^15,^^41^ and internationally.^1,19,42^ According to the WHO, only 12% of PC need is currently being met worldwide, and the need is expected to almost double by 2060.^43^ Additionally, access to inpatient PC varies, possibly due to barriers including sociodemographic and health-related factors, attitudes and beliefs, and insufficient resources.^44^ Possible consequences of insufficient PC competence range from unnecessary transitions between healthcare settings to avoidable human suffering.^20,45,46^ Therefore, ensuring a high level of competence promotes both the quality and cost-effectiveness of the healthcare system and the humane treatment of the patient and their next of kin.

Finland is currently adopting a three-tier model of PC services, in which services are divided into basic and specialised levels, each with their own responsibilities and requirements.^9,47^ PC and EOL care nursing is also included in a proposal for national clinical nursing specialties.^48^ From the perspective of the nursing profession in particular, these reforms present the need to examine whether the competence level of healthcare professionals working in various healthcare settings on different tiers of the service system meets the requirements set to the care they are providing, and whether their competence is at a level expected of a clinical nursing specialty. PC competence is thus a topical concern nationally in Finland, as well as on a global scale.

### 1.1. Objective

The objective of this study was to describe the self-assessed PC competence of healthcare professionals working in a university hospital, and to study which background factors are associated with it. This was done in order to identify areas of PC requiring improvement and further education in order to enhance the PC competence of healthcare professionals, and thus to improve not only the quality of care but also the quality of life of both patients and their next of kin.

### 1.2. Research questions

The following research questions were set for the quantitative and qualitative data, respectively:

1. What do healthcare professionals in a university hospital rate their PC competence? (*Quantitative data*)
2. Which background factors are associated with the self-assessed PC competence of healthcare professionals in a university hospital? (*Quantitative data*)
3. How do healthcare professionals in a university hospital describe their PC competence? (*Qualitative data*)

## 2. Methods

### 2.1. Study design

This study was conducted using an online survey in which participants were asked to rate their self-assessed competence in PC. A mixed methods approach with a convergent design was used to illustrate the quantitative ratings with qualitative assessments in the respondents’ own words and to offer a comprehensive view of the topic at hand.^49^ The reporting adhered to the STROBE checklist^50^ (Appendix 1) concerning the quantitative data and the SRQR checklist^51^ (Appendix 2) concerning the qualitative data. The study was conducted between September and December 2024.

### 2.2. Setting and participants

The study was conducted in one university hospital in Finland. The hospital was chosen because it is located in a large wellbeing services county where morbidity is high according to national morbidity indices,^52^ meaning that there is considerable need for PC and the setting was therefore considered to provide relevant data. Additionally, the researchers’ knowledge of and familiarity with the existing research infrastructure in the hospital contributed to the choice of setting. The target group consisted of healthcare professionals working with potential recipients of PC or EOL care. The inclusion criteria were a profession and a ward or unit in which the PC competence described by THL^15–17^ may be relevant; there was no need for exclusion criteria within this group. The survey was thus directed to nurses (registered nurses, midwives, public health nurses, practical nurses), emergency medical technicians (EMT), physical therapists, occupational therapists, and physicians, working in non-psychiatric specialised healthcare, rehabilitation, and emergency care services. Additionally, open options were provided for both profession and ward or unit, in case the participant had a different job title but otherwise met the inclusion criteria, since there is considerable heterogeneity in terms of titles in nursing, for instance.^53,54^

### 2.3. Data collection

The online survey was created using the survey platform Webropol. An invitation to participate, including a link to the survey, was delivered by a contact person to ward managers of units that met the inclusion criteria, who then distributed it to healthcare professionals. Data collection took place between November and December 2024, with reminders sent after 2 and 4 weeks. To obtain responses from as wide a range of professions and wards or units as possible, the sample size was not predetermined. The survey was conducted in Finnish; all excerpts from the scale and citations from the responses have been translated into English for this article by a member of the research team.

### 2.4. Questionnaire

The questionnaire contained background questions (n=13) on ward or unit, profession, education, work experience in healthcare overall and in PC specifically, supplementary education received regarding PC, and experiences regarding implementing PC in the participant’s place of work. The instrument for self-assessed PC competence consists of seven domains or quality areas (QAs) based on the QAs described in the THL quality handbook,^16^ containing a total of 30 items: “Living will and patient-centred work” (4 items), “Role of the patient’s next of kin and their interaction with the personnel” (4 items), “Advance care plan and promise of care continuity” (7 items, 2 of which are directed at all respondents, 2 at nurses only, and 3 at physicians only), “Ensuring competence” (4 items), “Availability of experts and securing the service chain” (4 items), “Other structural factors and assistive devices” (3 items), and “In the event of and after death” (4 items).

In the questionnaire, the participants rate their self-assessed competence for each item using a 6-point Likert-type scale (1=Poor, 2=Moderate, 3=Good, 4=Very good, 5=Excellent; 0=Not applicable to my job description). Participants are also asked to choose the ones out of the seven QAs where they feel the need for more education, with the ability to choose as many as they consider applicable. Finally, there are two open-ended questions: one for general thoughts on the respondent’s PC competence, and another for feedback regarding the questionnaire.

The instrument was developed for this study based on the THL quality handbook,^16^ with input from PC experts belonging to the research group. Although validation of the instrument was not among the study’s stated aims, and the instrument was not conclusively validated, validity and reliability were tested to enhance the overall reliability of the study. The content validity of the instrument was evaluated by a panel of experts (n=6), consisting of nurses and physicians who need PC competence in their work, who rated the items for relevance and clarity. An item-level content validity index (I-CVI) of 0.78 or higher has been suggested to indicate good content validity^55^; for this scale, the limit set for acceptable I-CVI was ≥0.8. Based on the evaluation, one item was removed, one was divided into two separate items, and two were slightly reworded, after which all items met this criterion.

Initial construct validity testing was performed using exploratory factor analysis with Kaiser-Meyer-Olkin (KMO) measure of sampling adequacy, Bartlett’s test of sphericity, principal axis factoring, and Varimax rotation. The KMO test gave a high value (.939), indicating adequate sampling, and the result of the Bartlett’s test was statistically significant (<.001).^56^ However, since the structure of the instrument was based on predetermined QAs, the instrument was left intact. Instrument reliability was tested by calculating Cronbach’s α values, which ranged from 0.896 to 0.953; values above 0.80 are considered indicative of a well-established instrument.^57^ Cronbach’s α for all 25 items directed at all respondents was 0.981 (Table 1).

**Table 1.** Quality areas and items included in the instrument, with means and Cronbach’s *α*′s for individual quality areas and for the entire instrument (translations for the purposes of this article only).

QA	Mean (minimum – maximum)	SD	Cronbach’s *α*
QA1: “Living will and patient-centred work”	12.55 (0 – 20)	4.88	.907
• QA1I1: “I am able to guide the patient on matters pertaining to living will”	2.29 (0 – 5)	1.48	​
• QA1I2: “I am able to take treatment intent into consideration when providing care, as required by my job description”	3.42	1.27	​
• QA1I3: “I am able to take treatment limitations [e.g. do-not-resuscitate (DNR) orders] into consideration when providing care, as required by my job description”	3.64	1.29	​
• QA1I4: “I am able to provide the patient with information regarding their medical state, as required by my job description”	3.20	1.46	​
QA2: “Role of the patient’s next of kin and their interaction with the personnel”	9.36 (0 – 20)	5.62	.947
• QA2I1: “I am able to support the next of kin of a patient receiving palliative or end-of-life care regarding their individual needs”	2.52 (0 – 5)	1.48	​
• QA2I2: “I am able to provide guidance to the next of kin of a patient receiving palliative or end-of-life care”	2.44	1.54	​
• QA2I3: “I am able to provide the next of kin of a patient receiving palliative or end-of-life care with pertinent information”	2.58	1.56	​
• QA2I4: “I am able to take the role and significance of guardianship into consideration in decision-making related to palliative care”	1.82	1.47	​
QA3: “Advance care plan and promise of care continuity” (items for all respondents)	4.51 (0 – 10)	3.17	.921
• QA3I1: “I am able to describe what an advance care plan should contain”	2.05 (0 – 5)	1.59	​
• QA3I2: “I am able to take the patient’s advance care plan into account when providing care”	2.46	1.70	​
QA3.1 (items for nurses only)	3.93 (0 – 10)	3.13	.917
• QA3I3: “I am able to recognise the need to update the patient’s advance care plan”	2.16 (0 – 5)	1.60	​
• QA3I4: “I am able to start the process of updating the patient’s advance care plan if needed”	1.76	1.66	​
QA3.2 (items for physicians only)	8.55 (0 – 15)	4.71	.896
• QA3I5: “I am able to recognise the need for an advance care plan”	2.85 (0 – 5)	1.60	​
• QA3I6: “I am able to draw up an advance care plan”	2.58	1.80	​
• QA3I7: “I am able to formulate medical treatment limitations and provide anticipatory care instructions”	3.12	1.76	​
QA4: “Ensuring competence”	12.00 (0 – 20)	5.38	.947
• QA4I1: “I am able to recognise symptoms exhibited by a patient receiving palliative or end-of-life care”	3.04 (0 – 5)	1.45	​
• QA4I2: “I am able to treat symptoms exhibited by a patient receiving palliative or end-of-life care by nonpharmacological means”	2.60	1.44	​
• QA4I3: “I am able to treat symptoms exhibited by a patient receiving palliative or end-of-life care by pharmacological means”	3.05	1.50	​
• QA4I4: “I am able to recognise the signs of approaching death”	3.31	1.40	​
QA5: “Availability of experts and securing the service chain”	10.12 (0 – 20)	5.76	.953
• QA5I1: “I am able to recognise the psychosocial needs of a patient receiving palliative or end-of-life care”	2.45 (0 – 5)	1.45	​
• QA5I2: “I am able to provide a patient receiving palliative or end-of-life care with expert support for psychosocial needs”	2.47	1.55	​
• QA5I3: “I am able to recognise the existential/spiritual needs of a patient receiving palliative or end-of-life care”	2.48	1.52	​
• QA5I4: “I am able to provide a patient receiving palliative or end-of-life care with expert support for existential/spiritual needs”	2.72	1.63	​
QA6: “Other structural factors and assistive devices”	7.99 (0 – 15)	4.52	.939
• QA6I1: “I am able to use assistive devices and equipment in palliative and end-of-life care”	2.59 (0 – 5)	1.61	​
• QA6I2: “I am able to recognise the patient’s individual needs and requests regarding matters such as privacy”	2.87	1.54	​
• QA6I3: “I am able to give the next of kin of a patient receiving palliative or end-of-life care the opportunity to participate in care, if the patient so wishes”	2.53	1.65	​
QA7: “In the event of and after death”	10.78 (0 – 20)	5.40	.924
• QA7I1: “I know how to act in the event of death”	3.16 (0–5)	1.52	​
• QA7I2: “I am able to support and console the bereaved”	3.02	1.47	​
• QA7I3: “I am able to guide the patient’s next of kin on practical matters following the patient’s passing, e.g. issues related to funeral arrangements”	2.32	1.54	​
• QA7I4: “I am able to direct the bereaved to appropriate sources of support”	2.28	1.46	​
Overall^a^	67.31 (0 – 125)	31.30	.981

Abbreviations: QA = quality area; SD = standard deviation.

^a^Items 3.3-3.7 have been excluded from the sum variables due to only applying to certain participants based on profession.

### 2.5. Data analysis

For the analysis, participants were grouped by profession (Nurses, Physicians, Other), and by ward or unit (General Medicine and Geriatrics; Oncology; Intensive Care, Anaesthesia, and Surgery; Internal Medicine; Gastroenterology and Sensory Organs; Other). Work experience, both in healthcare overall and in PC specifically, was stratified into three groups: “Less than two years of experience”, “2 to 10 years of experience”, and “More than 10 years of experience”.

Descriptive analyses included frequencies and percentages for categorical variables (profession; ward or unit; work experience in healthcare as a whole, and in PC specifically; education; awareness and education regarding PC skills). For the purposes of analysis, the ordinal data obtained from the Likert-type scale used for the items were treated as interval data^58^; PC self-competence is presented as means for each of the seven QAs and for the entire instrument. When examining gerontological nursing competence on a five-point Likert scale, Suonnansalo et al.^59^ among others have used a three-level classification scheme of “Very good”=3.5–5, “Good”=2.5–3.499, and “Fair”=1–2.499. To facilitate comparing the results of this study to existing research regarding healthcare competence, the same scheme was applied here.

Regarding factors associated with self-assessed PC competence, comparisons between groups (professions, wards or units, work experience in healthcare overall and in PC specifically) were conducted using the one-way analysis of variance (ANOVA) test (normal distribution), and the Kruskal-Wallis test for more than two independent groups (skewed distribution). Normality was determined using graphical methods (box plots and histograms), with the guidance of a statistician.^60^ Pairwise comparisons were conducted using post-hoc tests: Games-Howell for normally distributed variables with different group sizes, and the Mann-Whitney U test with Bonferroni correction in the case of skewed distribution.^61,62^

The two-open ended questions yielded a total of 51 responses; additionally, the five open-ended responses to the question about the area in which the participant feels the need for more education were included, since they contained pertinent information in terms of the research question. The open-ended responses (n=56) were analysed using inductive content analysis, consisting of several phases: firstly, the data were reduced into codes (n=106); secondly, the data were grouped based on similarities and subcategories were formed (n=8); thirdly, the subcategories were used to form main categories (n=3) to provide answers to research questions,^63,64^ with response to the question as the unit of analysis.^65^ The analysis was performed concurrently with the quantitative analysis by one member of the research team with practical PC experience, with support from research team members with extensive experience in conducting content analysis.

The data analysis was conducted using IBM SPSS Statistics (Version 29.0.1.0). The cutoff value used for statistical significance was *p*<.05.^66^

### 2.6. Ethical considerations

The appropriate research permit was obtained from the wellbeing services county in question prior to data collection. According to Finnish legislation and guidelines regarding medical research and research with human participants, since the research design did not contain elements such as deviating from the principle of informed consent or causing a threat to the participants’ safety, ethical review by an ethics committee was not required.^67^ Informed consent was obtained from all participants at the beginning of the questionnaire, and information regarding the confidentiality and anonymity of the survey was provided before responding. The participants were informed that participation was voluntary, and no compensation was offered for participating. Before commencing data collection, risk assessment regarding data protection and information security was conducted, and the data were stored and handled accordingly.^68,69^ Good ethical code of conduct, such as honesty, respect, and responsibility, was observed by maintaining quality, openness, impartiality, and collegiality throughout the process.^70,71^

## 3. Results

### 3.1. Respondent characteristics

A total of 100 participants responded to the questionnaire, 62% of whom were nurses (registered nurse, midwife, or practical nurse), 27% physicians, and 11% representatives of other professions. In terms of units and specialties, the largest group represented was General Medicine and Geriatrics (28%), followed by Oncology (including a palliative inpatient ward) (19%), and Intensive Care, Anaesthesia, and Surgery (17%). The median for overall work experience in healthcare was 11 to 15 years; for work experience related to PC, the median was 2 to 5 years. Out of all participants, 23% reported receiving enough supplementary training and education regarding PC, whereas 34% found the amount of supplementary training insufficient. When asked whether the participant considers PC competence important in their work, 75% either completely or partly agreed (Table 2).

**Table 2.** Background statistics of participants.

Variable	*n* (%)	[95% CI]
*Profession*		
• Nurse	62 (62)	[52.3, 71.1]
• Physician	27 (27)	[19.0, 36.3]
• Other	11 (11)	[6.0, 18.2]
*Work unit*		
• General Medicine and Geriatrics	28 (28)	[19.9, 37.3]
• Oncology (including Palliative inpatient)	19 (19)	[12.3, 27.5]
• Intensive Care, Surgery, and Anaesthesia	17 (17)	[10.6, 25.2]
• Internal Medicine	16 (16)	[9.8, 24.1]
• Gastrology and Sensory Organs	10 (10)	[5.3, 17.0]
• Other	10 (10)	[5.3, 17.0]
*Education* ^a^		
• Vocational	24	​
• Bachelor’s degree, University of Applied Sciences	65	​
• Master’s degree, University of Applied Sciences	9	​
• Bachelor’s degree	4	​
• Master’s degree/Licentiate of Medicine degree	24	​
• Doctoral degree	9	​
• Other	8	​
*Work experience in healthcare*		
• Less than 2 years	4 (4)	[1.4, 9.2]
• 2 to 5 years	11 (11)	[6.0, 18.2]
• 6 to 10 years	21 (21)	[13.9, 29.7]
• 11 to 15 years	17 (17)	[10.6, 25.2]
• More than 15 years	47 (47)	[37.4, 56.8]
*Work experience in palliative care*		
• No experience	18 (18)	[11.4, 26.4]
• Less than 2 years	21 (21)	[13.9, 29.7]
• 2 to 5 years	17 (17)	[10.6, 25.2]
• 6 to 10 years	20 (20)	[13.1, 28.6]
• 11 to 15 years	6 (6)	[2.5, 11.9]
• More than 15 years	18 (18)	[11.4, 26.4]
*I have obtained the THL End-of-life care passport* ^72^		
• Yes	14 (14)	[8.3, 21.8]
• No	86 (86)	[78.2, 91.7]
*I have received information about symptom management in palliative care or end-of-life care as part of my orientation*		
• Yes	46 (46)	[36.5, 55.8]
• No	45 (45)	[35.5, 54.8]
• Unsure	9 (9)	[4.5, 15.8]
*I am familiar with the consultation practices of my organisation regarding palliative care and end-of-life care*		
• Yes	59 (59)	[49.2, 68.3]
• No	34 (34)	[25.3, 43.6]
• Unsure	7 (7)	[3.2, 13.3]
*I am able to consult different parties on matters concerning a patient receiving palliative care or end-of-life care, if needed*		
• Yes	72 (72)	[62.7, 80.1]
• No	17 (17)	[10.6, 25.2]
• Unsure	11 (11)	[6.0, 18.2]
*I am able to receive clinical supervision related to palliative care or end-of-life care, if needed*		
• Yes	34 (34)	[25.3, 43.6]
• No	35 (35)	[26.2, 44.7]
• Unsure	31 (31)	[22.6, 40.5]
*I need palliative care and end-of-life care competence in my work*		
• Completely agree	56 (56)	[46.2, 65.4]
• Partly agree	19 (19)	[12.3, 27.5]
• Neither agree nor disagree	2 (2)	[0.4, 6.3]
• Partly disagree	17 (17)	[10.6, 25.2]
• Completely disagree	6 (6)	[2.5, 11.9]
*I encounter patients receiving palliative care or end-of-life care in my work*		
• Every day	20 (20)	[13.1, 28.6]
• Every week	20 (20)	[13.1, 28.6]
• Every month	23 (23)	15.6, 31.9]
• A few times per year	22 (22)	[14.8, 30.8]
• Less often or never	15 (15)	[9.0, 23.0]
*I have received supplementary training related to palliative care and end-of-life care* ^a^		
• Regularly	10	[5.3, 17.0]
• Enough	23	[15.6, 31.9]
• Not enough	34	[25.3, 43.6]
• Never	40	[30.8, 49.8]

Abbreviations: CI = Confidence interval.

^a^Option to select all that apply.

### 3.2. Self-assessed PC competence

Means are reported for each of the seven QAs and for the entire instrument (Table 1). The overall mean is reported for each individual item from highest to lowest, applying the classification scheme of “Very good”=3.5–5, “Good”=2.5–3.499, and “Fair”=1–2.499^59^ (Table 3).

**Table 3.** Means for individual items, in order of highest to lowest mean, applying the “Very good” – “Good” – “Fair” classification scheme^59^ (translations for the purposes of this article only).

Item	Mean^a^	SD
QA1I3: “I am able to take treatment limitations [e.g. do-not-resuscitate (DNR) orders] into consideration when providing care, as required by my job description”	3.64 (*Very good*)	1.29
QA1I2: “I am able to take treatment intent into consideration when providing care, as required by my job description”	3.42 (*Good*)	1.27
QA4I4: “I am able to recognise the signs of approaching death”	3.31	1.40
QA1I4: “I am able to provide the patient with information regarding their medical state, as required by my job description”	3.20	1.46
QA7I1: “I know how to act in the event of death”	3.16	1.52
QA3I7^b^:“I am able to formulate medical treatment limitations and provide anticipatory care instructions”	3.12	1.76
QA4I3: “I am able to treat symptoms experienced by a patient receiving palliative or end-of-life care by pharmacological means”	3.05	1.50
QA4I1: “I am able to recognise symptoms in a patient receiving palliative or end-of-life care”	3.04	1.45
QA7I2: “I am able to support and console the bereaved”	3.02	1.47
QA6I2: “I am able to recognise the patient’s individual needs and requests regarding matters such as privacy”	2.87	1.54
QA3I5^b^: “I am able to recognise the need for an advance care plan”	2.85	1.60
QA5I4: “I am able to provide a patient receiving palliative or end-of-life care with expert support for existential/spiritual needs”	2.72	1.63
QA4I2: “I am able to treat symptoms experienced by a patient receiving palliative or end-of-life care by nonpharmacological means”	2.60	1.44
QA6I1: “I am able to use assistive devices and equipment in palliative and end-of-life care”	2.59	1.61
QA2I3: “I am able to provide the next of kin of a patient receiving palliative or end-of-life care with pertinent information”	2.58	1.56
QA3I6^b^:“I am able to draw up an advance care plan”	2.58	1.80
QA6I3: “I am able to give the next of kin of a patient receiving palliative or end-of-life care the opportunity to participate in care, if the patient so wishes”	2.53	1.65
QA2I1: “I am able to support the next of kin of a patient receiving palliative or end-of-life care regarding their individual needs”	2.52	1.48
QA5I3: “I am able to recognise the existential/spiritual needs of a patient receiving palliative or end-of-life care”	2.48 (*Fair*)	1.52
QA5I2: “I am able to provide a patient receiving palliative or end-of-life care with expert support for psychosocial needs”	2.47	1.55
QA3I2: “I am able to take the patient’s advance care plan into account when providing care”	2.46	1.70
QA5I1: “I am able to recognise the psychosocial needs of a patient receiving palliative or end-of-life care”	2.45	1.45
QA2I2: “I am able to provide guidance to the next of kin of a patient receiving palliative or end-of-life care”	2.44	1.54
QA7I3: “I am able to guide the patient’s next of kin on practical matters following the patient’s passing, e.g. issues related to funeral arrangements”	2.32	1.54
QA1I1: “I am able to guide the patient on matters pertaining to living will”	2.29	1.48
QA7I4: “I am able to direct the bereaved to appropriate sources of support”	2.28	1.46
QA3I3^c^: “I am able to recognise the need to update the patient’s advance care plan”	2.16	1.60
QA3I1: “I am able to describe what an advance care plan should contain”	2.05	1.59
QA2I4: “I am able to take the role and significance of guardianship into consideration in decision-making related to palliative care”	1.82	1.47
QA3I4^c^:“I am able to start the process of updating the patient’s advance care plan if needed”	1.76	1.66

Abbreviations: QA = quality area; I = item; SD = standard deviation; PC = palliative care.

Scale used in questionnaire: 1 = Poor, 2 = Moderate, 3 = Good, 4 = Very good, 5 = Excellent, 0 = Not applicable to my job description.

^a^Min 0, max 5 for all items.

^b^Item directed at physicians only.

^c^Item directed at nurses only.

When asked to indicate the area(s) in which the participant felt the need for more education, QA3 and QA5 were the most common answers (47% each), while QA6 and QA7 were the least common (37% each) (Table 4). Additionally, topics named in the open-ended responses included more education on correctly timed treatment decisions, the social and financial benefits for a patient receiving PC, parties to consult regarding PC in the wellbeing services county in question, and the THL “End-of-life care passport” (*Saattohoitopassi*), an online course available between 2021 and 2025 that covered the basics of EOL care.^72^

**Table 4.** Quality areas in which participants felt the need for more education.

QA	*n* (%)	[95% CI]
QA1: “Living will and patient-centred work”	40 (40)	[30.8, 49.8]
QA2: “Role of the patient’s next of kin and their interaction with the personnel”	45 (45)	[35.5, 54.8]
QA3: “Advance care plan and promise of care continuity”	47 (47)	[37.4, 56.8]
QA4: “Ensuring competence”	39 (39)	[29.9, 48.8]
QA5: “Availability of experts and securing the service chain”	47 (47)	[37.4, 56.8]
QA6: “Other structural factors and assistive devices”	37 (37)	[28.0, 46.7]
QA7: “In the event of and after death”	37 (37)	[28.0, 46.7]
Other topic	5 (5)	[1.9, 10.6]
None of the above	18 (18)	[11.4, 26.4]

Abbreviations: QA = quality area, CI = Confidence interval.

Option to select all that apply.

### 3.3. Background factors associated with self-assessed PC competence

Comparisons between groups were based on the mean score for each QA separately (in QA3, items 3.3–3.7 were excluded, due to only being available to some of the participants based on profession). Groups compared consisted of professions, wards or units, and work experience in healthcare overall and in PC specifically, respectively (Table 5). Due to the small size of the group with less than two years of work experience in healthcare overall (n=4), the values for the group are not reported.

**Table 5.** Mean scores for quality areas and overall mean by profession, work unit, work experience in healthcare, and work experience in PC.

QA (minimum score – maximum score)	By profession				By work unit							By work experience in healthcare			By work experience in PC			
	Nurses (*n* = 62) mean (SD)	Physicians (*n* = 27) mean (SD)	Other (*n* = 11) mean (SD)	*p*-value	General medicine and geriatrics (*n* = 28) mean (SD)	Oncology (*n* = 19) mean (SD)	Intensive care, surgery, and anaesthesia (*n* = 17) mean (SD)	Internal medicine (*n* = 16) mean (SD)	Gastrology and sensory organs (*n* = 10) mean (SD)	Other (*n* = 10) mean (SD)	*p*-value	2–10 years (*n* = 32) mean (SD)	>10 years (*n* = 64) mean (SD)	*p*-value	<2 years (*n* = 39) mean (SD)	2–10 years (*n* = 37) mean (SD)	>10 years (*n* = 24) mean (SD)	*p*-value
QA1: “Living will and patient-centred work” (0 – 20)	12.52 (4.16)	15.00 (3.70)	6.73 (6.42)	**<.001**	13.68 (3.95)	14.21 (4.10)	9.76 (6.14)	12.19 (2.90)	14.20 (4.47)	9.90 (6.72)	.051	13.59 (3.75)	12.03 (5.42)	.412	9.85 (5.45)	14.86 (3.06)	13.38 (4.16)	**<.001**
QA2: “Role of the patient’s next of kin and their interaction with the personnel” (0 – 20)	9.40 (5.38)	11.07 (5.53)	4.91 (5.17)	**.008**	10.75 (5.10)	11.84 (5.75)	6.18 (6.02)	8.00 (3.86)	9.70 (5.95)	8.00 (5.87)	**.038**	10.22 (4.99)	9.03 (6.00)	.469	6.00 (5.24)	11.73 (4.82)	11.17 (4.73)	**<.001**
QA3: “Advance care plan and promise of care continuity” (0 – 10)^a^	4.16 (3.05)	6.30 (2.73)	2.09 (2.74)	**<.001**	5.86 (2.61)	5.68 (3.38)	2.18 (2.65)	3.31 (2.27)	4.80 (3.55)	4.10 (3.41)	**.002**	4.81 (2.95)	4.42 (3.31)	.686	2.97 (2.92)	5.70 (2.92)	5.17 (2.99)	**<.001**
QA4: “Ensuring competence” (0 – 20)	12.47 (4.80)	13.15 (4.30)	6.55 (7.79)	**.001**	13.21 (4.38)	13.58 (5.41)	9.76 (6.41)	12.50 (2.73)	10.90 (5.93)	9.70 (7.41)	.244	12.38 (5.29)	11.75 (5.55)	.860	8.44 (5.92)	14.49 (3.25)	13.96 (3.85)	**<.001**
QA5: “Availability of experts and securing the service chain” (0 – 20)	10.44 (5.43)	10.59 (5.36)	7.18 (7.93)	.200	11.64 (4.52)	12.26 (6.12)	7.35 (6.00)	8.38 (5.15)	10.40 (5.34)	9.00 (7.32)	.095	11.06 (5.90)	9.73 (5.84)	.323	6.49 (5.57)	12.73 (4.58)	12.00 (4.64)	**<.001**
QA6: “Other structural factors and assistive devices” (0 – 15)	8.92 (4.12)	7.44 (4.37)	4.09 (5.15)	**.003**	9.89 (3.80)	8.68 (5.27)	7.18 (4.57)	6.94 (3.73)	5.80 (4.16)	6.60 (5.06)	**.041**	8.69 (4.28)	7.58 (4.75)	.598	5.10 (4.38)	10.08 (3.70)	9.46 (3.40)	**<.001**
QA7: “In the event of and after death” (0 – 20)	11.81 (4.73)	11.26 (4.83)	3.82 (5.56)	**<.001**	12.71 (4.34)	12.26 (6.57)	9.88 (6.05)	8.81 (4.40)	9.70 (3.37)	8.30 (6.02)	**.040**	11.84 (5.12)	10.27 (5.65)	.506	7.51 (5.32)	13.86 (3.60)	11.33 (4.98)	**<.001**
Overall (0 – 125)^a^	69.71 (28.19)	74.81 (28.31)	35.36 (38.16)	**<.001**	77.75 (25.97)	78.53 (33.42)	52.29 (34.43)	60.13 (19.86)	65.50 (29.19)	55.60 (40.28)	**.027**	72.59 (28.78)	64.81 (33.26)	.426	46.36 (31.02)	83.46 (22.62)	76.46 (23.97)	**<.001**

Abbreviations: PC = palliative care; QA = quality area; SD = standard deviation.

One-way ANOVA. 95% CI (confidence interval) (multiple comparisons), Games-Howell (post-hoc pairwise comparisons); Independent samples Kruskal-Wallis test (multiple comparisons); Mann-Whitney U test (two-sided; post-hoc pairwise comparisons). For pairwise comparisons, significance values have been adjusted by the Bonferroni correction for multiple tests. Statistically significant *p*-values (<.05) **in bold**; *p*-values from .000 up to .001 given and reported as <.001.

^a^Items 3.3-3.7 have been excluded from the sum variables due to only applying to certain participants based on profession.

Comparing by profession, physicians’ and nurses’ self-assessed competence was considerably higher than that of other professions, the differences being statistically significant regarding most QAs; the overall average was 69.71 (out of 125) for nurses (SD 28.19), 74.81 for physicians (SD 28.31), and 35.36 for other professions (SD 38.16). In terms of wards or units, the ones mostly scoring the highest were Oncology (overall average 78.53, SD 33.42) as well as General Medicine and Geriatrics (overall average 77.75, SD 25.97), but only some of the differences concerning specific QAs were statistically significant. Work experience in PC affected self-assessed competence, with those who had 2–10 years of experience scoring the highest (overall average 83.46, SD 22.62), whereas differences based on work experience in healthcare overall were not statistically significant.

Due to the heterogeneity of the respondents in terms of profession, meaningful comparisons based on education were unfeasible.

### 3.4. Healthcare professionals’ descriptions of self-assessed PC competence

**Figure 1.**
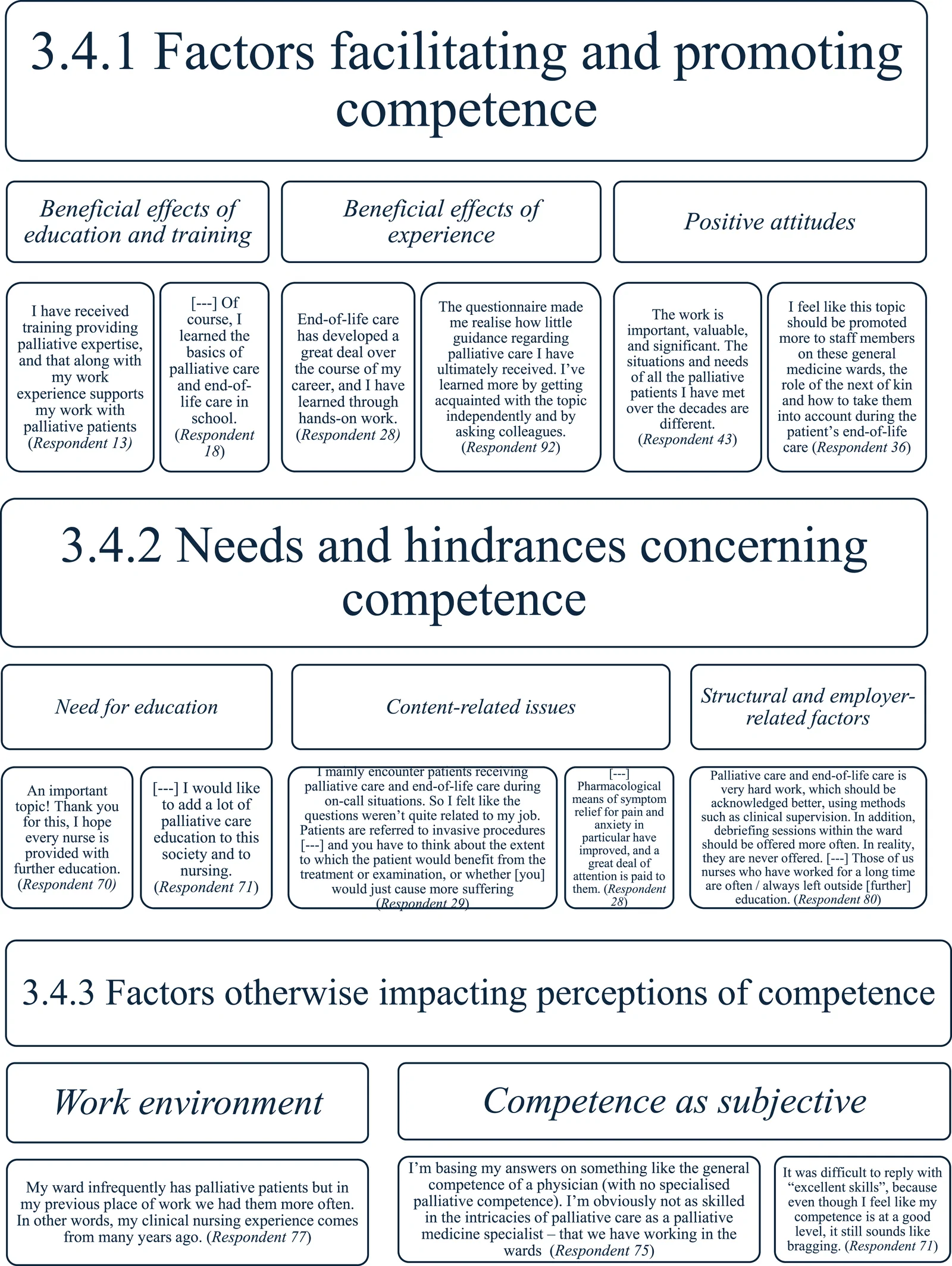
Healthcare professionals’ descriptions of self-assessed palliative care competence. Citations from the open-ended responses (translated from Finnish for this article).

Inductive content analysis revealed three main categories: *Factors facilitating and promoting competence*, *Needs and hindrances concerning competence*, and *Factors otherwise impacting perceptions of competence*. Citations from the responses are provided in Figure 1.

#### 3.4.1. Factors facilitating and promoting competence

Three main categories of factors facilitating and promoting the realisation of PC competence emerged. *Beneficial effects of education and training* included basic studies qualifying the participant to work in healthcare, specialty training and education concerning PC (such as that of a PC specialist), and voluntary courses focusing on PC.

*Beneficial effects of experience* meant becoming more competent in PC by encountering PC in one’s work. Two participants also mentioned actively developing their competence by getting acquainted with the topic on their own, as opposed to any formal training.

*Positive attitudes* appeared as statements affirming the significance of PC competence and wishing for greater awareness of its importance.

#### 3.4.2. Needs and hindrances concerning competence

The participants named several issues concerning needs related to PC competence. *Need for education* entailed a desire to receive more education, both personally and in the field of healthcare, as well as in society in general.

*Content-related issues* comprised needs concerning various specific topics. Competence regarding treatment decisions and limitations appeared most frequently, but areas including social benefits, pharmacological symptom management, and the needs of special groups such as children and the patient’s next of kin were mentioned as well.

*Structural and employer-related factors* included resources for promotion of PC competence, equal further education opportunities for all staff members (e.g. regardless of how long they have worked in the ward), and sufficient orientation to PC.

#### 3.4.3. Factors otherwise impacting perceptions of competence

In addition to factors clearly promoting competence or concerning perceived needs, the participants mentioned factors that otherwise impact perceptions or expectations regarding competence, including the participant’s *Work environment* and the idea of *Competence as subjective*.

*Work environment,* i.e. the kind of unit in which the participant worked, altered the participant’s view of their need for or level of PC competence, based on whether patients with PC needs were encountered there. *Competence as subjective,* addressed by individual participants, meant contrasting one’s self-assessment of competence on the competence of others: whether comparing to the average professional or to someone specialised in PC was seen to affect how to rate oneself, as was uncertainty regarding whether one can call one’s own competence “excellent” in the first place.

## 4. Discussion

Although previous studies have examined PC competence both in different contexts in Finland^13,22,25–29^ and in hospital settings elsewhere,^73–75^ this is the first study to our knowledge to specifically focus on self-assessed PC competence in a Finnish university hospital. The results provide much-needed information regarding the state of PC competence in Finland from the perspective of those whose work does, or at least may, involve PC. The quantitative data provided information on perceived levels of competence in various areas, and showed correlations between competence and certain background factors. The qualitative data offered descriptions of PC competence in the respondents’ own words, as well as further insight into what might promote or hinder competence, thus deepening the understanding of self-assessed PC competence gained from the quantitative results. The study had a convergent design, where the quantitative and qualitative data were collected at the same time but were analysed separately and independently from one another.^49^ This both enabled deeper and more nuanced reflection of the implications of the quantitative findings and placed the personal observations expressed in the qualitative data into a quantifiable context.

Most of the items (17 out of 30) were rated “Good”^59^ among all respondents; additionally, one was even rated “Very good,” the highest level available. In previous studies focusing on hospitals,^73–75^ PC competence has varied. Among ICU nurses, nearly 80% of the respondents were in the “low” and “medium” groups concerning overall core competency in PC^75^; 60% of our items were rated “Good” or “Very good,” suggesting a more positive assessment regarding competence, although areas requiring improvement also emerged. As in previous research,^19^ most respondents considered PC competence necessary in their work, which was also reflected in the responses to the open-ended questions.

The QA with the highest overall mean score, “Living will and patient-centred work,” concerned providing appropriate clinical care, information, and guidance. For individual items, many of those with the highest mean scores concerned practical skills. These included taking treatment limitations into consideration when treating or caring for the patient (the only item rated “Very good”), recognising the signs of approaching death, and knowing how to act in the event of a patient’s death. In the open-ended responses, participants brought up the importance of appropriate and correctly timed treatment decisions and limitations. As competence regarding following treatment limitations in clinical care was rated high, the importance of making sure that limitations are set appropriately should be emphasised while providing PC.

The domain rated the lowest was “Advance care plan and promise of care continuity”. Individual items with the lowest scores included those involving advance care planning and taking the role and significance of guardianship into consideration in decision-making. Healthcare workers might benefit from more education on advance care planning and on the contents of an advance care plan – an area also identified as important in previous research.^22^ Likewise, more education for healthcare workers regarding social welfare and benefits may be warranted, which was expressed in the open-ended responses as well.

When comparing professional groups, the “Other” group assessed their competence to be at a lower level than did either nurses or physicians. While the small size and heterogeneity of the “Other” group in this study prevents drawing any far-reaching conclusions, it suggests a need for further research on PC competence among different professions, especially those other than nurses or physicians, such as physical and occupational therapists. This is especially important since previous research has noted both the importance of profession-specific competence in PC and the need for further research on the topic.^76^

In the case of work units, General Medicine and Geriatrics as well as Oncology mostly scored the highest. This possibly stems from the needs of the patients requiring the staff to possess this kind of competence, the nature of everyday work in the wards providing opportunities to improve competence, or perhaps a combination of the two. Different requirements concerning PC competence based on work environment were also recognised in the open-ended responses. Differences between wards or units require further research; a larger sample size would also enable comparisons between professionals working in different units or specialties.

There were statistically significant differences by work experience in PC specifically, with competence at its highest in the “2 to 10 years of experience” group, but not by overall work experience in healthcare, which was partly in line with previous studies^26,^^30^ and highlights the need for specialised PC education. Interestingly, competence was not rated highest among those with the most experience. In the open-ended responses, a respondent stated that those who have worked in the ward the longest are consistently left outside further education opportunities. Perhaps this middle group gains the simultaneous benefits of both practical work experience and supplementary education better than do either newcomers or long-term staff members. To improve competence, further education should be provided equally to all members of staff. Differences in self-assessed competence based on previous PC experience, as well as the impact of further education opportunities and education received, merit further research.

Many of the skills covered in the questionnaire involve the work of several professions, with different roles for each participant; some of the skills with lower scores (such as skills related to guardianship) concern several professional groups. Moreover, the effects – both positive and negative – of the competence of other professionals on one’s own work were discussed in the open-ended responses: for example, personal competence was credited to working as part of a skilled multiprofessional team, but the competence of physicians regarding treatment decisions was also considered to impact nurses’ ability to provide good PC, either promoting or hindering it. The importance of well-functioning multiprofessional cooperation^29,76^ should be acknowledged and examined in more depth in future studies.

The results of this study can be used to provide professionals with support such as clinical supervision and education interventions on topics with the greatest need for improvement, and to develop PC education to address these questions more extensively during medical or nursing studies. Nurse managers and other healthcare supervisors can use the results as guidance on allocating personnel resources to tasks where, for example, competence is enhanced by working in a multiprofessional team or with an experienced colleague. The results can also serve as a reminder for those in supervisor positions to provide further education opportunities equally to all members of staff.

### 4.1. Strengths and limitations

Ethical conduct^70^ and careful and open reporting of the research process and results was maintained to ensure trustworthiness throughout. Since high-quality PC involves a multiprofessional approach,^29^ the study included different professions, which helps offer a varied overview of the current state of PC.

The publication of the THL quality recommendations and handbook,^15,16^ developed with the Finnish healthcare context in mind, introduced the need to develop an instrument for measuring competence in terms of the national quality criteria, and the instrument used in this study was developed to respond to the unique needs identified nationally. While the validity and reliability of some instruments measuring nursing self-competence has been found lacking particularly in terms of PC,^36^ some scales especially developed to assess PC competence have been validated for use in different countries and for various patient categories.^36–40^ However, unlike previous instruments, this one was developed specifically for the current Finnish context to respond to the need to measure PC competence in Finland based on national quality criteria, which strengthened the validity of the study.

While the instrument used has not been formally validated, the study also provided an opportunity to test its validity and reliability. The content validity of the instrument was enhanced by the evaluation of the panel of experts, and the I-CVI was found to be good; construct validity and instrument reliability were also tested and revealed to be good. The instrument was pilot-tested by members of the research group (n=2), but not by a separate pilot group. However, the instrument reliability and content and construct validity of the instrument support developing it further, so that after validation, it could be used in future studies as well. Since the electronic survey tool used prevents participants from returning an incomplete form, there were no missing responses in the data. While the researchers involved in the analysis were familiar with the hospital in question, the responses were anonymous, which enhanced objectivity.

The study had some limitations as well. The inclusion of several professional groups may also be seen as a weakness, as some questions might have been more relevant to certain professional groups than to others. Future studies on PC competence in a university hospital setting might concentrate on one professional group at a time. However, the approach taken here enabled comparison between various professions, which provided valuable new information and identified potential topics for further research. The instrument was based on Finnish guidelines regarding PC, so neither the results nor the instrument may be directly transferable to other countries. At the same time, the national guidelines take international recommendations, such as those by the European Association for Palliative Care (EAPC),^77^ into consideration; this increases the applicability of the instrument to other countries, with appropriate revisions depending on the healthcare system in the country in question.

No power analysis was done for sample size calculation, since the decision was made to not predetermine the sample size. The questionnaire was in Finnish only, so the language barrier may have served as an implicit exclusion criterion. While the validity of the Finnish original seems promising, using the instrument in other languages would require validation with adaptations as needed. There is the possibility of selection bias, as those who would rate their competence the lowest might be the least likely to respond. According to the open-ended responses, rating one’s competence “excellent” might feel like bragging, even if accurate, suggesting possible cultural and sociological factors affecting giving oneself a high score.^78,79^ Members of the research team had firsthand experience of working in PC, which might have made it more difficult to maintain objectivity towards the topic, although it did also serve to enhance validity.

One limitation of the study was lack of clarity regarding the response rate. Out of 5,000 healthcare professionals fulfilling the inclusion criteria in the applicable wards and units, only 100 responded, which would amount to a response rate of 2%; however, since the invitation was distributed via several contact persons using different mailing lists, ascertaining the exact number of potential respondents reached proved impossible. As a result, the response rate cannot be reported with any certainty. In addition, online surveys generally have low response rates.^80,81^ Furthermore, there are currently several reforms and other projects taking place in the Finnish healthcare system that may have hindered the possibility to participate in surveys even further.

Since the number of potential respondents actually reached or the reason for non-participation could not be ascertained, the role of nonresponse bias could not be examined reliably. The challenges regarding ascertaining who received the invitation also made bias analysis largely unfeasible. A future study with a higher, more precise response rate might shed more light on statistical differences between professional groups and specialties. However, this study provided much-needed information about self-assessed PC competence from several professional groups and across specialties, allowed the professionals to express their views in their own words, and served to test the validity and reliability of the newly developed instrument. Although a higher response rate would have improved generalisability, the respondents represented a variety of professions and wards or units. The survey therefore captured a sample representing most, albeit not all, professions and units in which the study was interested.

Since the study focused on self-assessment, the responses might not reflect the objective reality of the need for or state of PC competence. Nevertheless, they represent the authentic views and attitudes of those with firsthand professional experience of PC, and thus offer valuable insight into what areas of PC professionals feel confident in, and where they see room for improvement. As Finland is moving towards a new way of organising PC services, there is a need to both identify strengths and weaknesses in professionals’ competence and monitor the development of competence. With validation, the instrument used here could be employed for that purpose in the future as well.

## 5. Conclusion

This mixed methods survey provided information about the self-assessed palliative care competence of healthcare professionals in a Finnish university hospital. The quantitative data showed what professionals rate their competence on a numerical scale, while the qualitative data gave them the opportunity to describe their competence and the factors contributing to it in more detail. Based on the results, healthcare professionals considered palliative care competence important, and overall, competence was assessed to be good. At the same time, the study revealed areas potentially in need of improvement and more education, both indirectly as lower scores for self-assessed competence and directly as comments provided by the participants.

The quality area with the highest overall mean score concerned providing appropriate clinical care, information, and guidance; the individual items that scored the highest involved practical skills such as taking treatment limitations into consideration in one’s work, recognising the signs of approaching death, and knowing how to act in the event of death. The items with the lowest scores, and the lowest-rated quality area, involved advance care planning, as well as questions such as those related to guardianship. In their open-ended responses, respondents discussed topics such as factors promoting or hindering competence, and specific questions on which further education is needed, shedding further light on what professionals consider to influence their palliative care competence.

In terms of further research, the instrument developed to measure self-assessment in this study was preliminarily tested for reliability and validity with promising results. While conclusive validation is needed, and use in different contexts would require adaptations, the tentative indications of reliability and validity suggest that the instrument could be used in further studies with a larger, more clearly delineated target group. This would enable examination of the perceived strengths and weaknesses of healthcare professionals, as well as differences between various groups, in more detail.

The results suggest that healthcare professionals feel the need for more education on some aspects of palliative and end-of-life care, especially regarding advance care planning, guardianship, and multiprofessional cooperation. They also consider this competence important, both in practical terms considering everyday work and because good palliative care is considered valuable. Responding to this need for more education is warranted from the perspective of both the quality of healthcare and the quality of life of patients receiving palliative care.

## Supplemental Material


Supplemental Material - Self-assessed palliative care competence of healthcare professionals in a Finnish university hospital: A mixed methods study
Supplemental Material for Self-assessed palliative care competence of healthcare professionals in a Finnish university hospital: A mixed methods study by Leena Vuolteenaho, Minna Hökkä, Eeva Rahko, Tarja Pölkki and Mira Rajala in Palliative Care and Social Practice.

Supplemental Material - Self-assessed palliative care competence of healthcare professionals in a Finnish university hospital: A mixed methods study
Supplemental Material for Self-assessed palliative care competence of healthcare professionals in a Finnish university hospital: A mixed methods study by Leena Vuolteenaho, Minna Hökkä, Eeva Rahko, Tarja Pölkki and Mira Rajala in Palliative Care and Social Practice.

Supplemental Material - Self-assessed palliative care competence of healthcare professionals in a Finnish university hospital: A mixed methods study
Supplemental Material for Self-assessed palliative care competence of healthcare professionals in a Finnish university hospital: A mixed methods study by Leena Vuolteenaho, Minna Hökkä, Eeva Rahko, Tarja Pölkki and Mira Rajala in Palliative Care and Social Practice.

## Data Availability

The data that support the findings of this study are available from the corresponding author upon reasonable request until the publication of the article, after which the data are destroyed as per the data management plan for this study.*
