# Supplementary material for: Self-assessed palliative care competence of healthcare professionals in a Finnish university hospital: A mixed methods study
Source: Palliat Care Soc Pract. 2026 Aug 25;20:26323524261480156. doi: 10.1177/26323524261480156 (PMC13519223; doi:10.1177/26323524261480156)
Supplement: Supplementary file 1 [file sj-pdf-1-pcr-10.1177_26323524261480156.pdf]

Appendix 1. STROBE checklist[50] for the article.

STROBE Statement—checklist of items that should be included in reports of observational studies

	Item No.	Recommendation	Page No.	Relevant text from manuscript
**Title and abstract**	1	(*a*) Indicate the study's design with a commonly used term in the title or the abstract	abstract	Mixed methods study
		(*b*) Provide in the abstract an informative and balanced summary of what was done and what was found	abstract	This mixed methods study was conducted in one university hospital in Finland in 2024…
**Introduction**				
Background/rationale	2	Explain the scientific background and rationale for the investigation being reported	1–2	Palliative care (PC) aims to improve the quality of life of patients with life-threatening illnesses…
Objectives	3	State specific objectives, including any prespecified hypotheses	2–3	The objective of this study was to describe the self-assessed PC competence... in order to identify areas of PC requiring improvement…
**Methods**				
Study design	4	Present key elements of study design early in the paper	3	This study was conducted using an online survey… A mixed methods approach with a convergent design was used to illustrate...
Setting	5	Describe the setting, locations, and relevant dates, including periods of recruitment, exposure, follow-up, and data collection	3–4	The study was conducted in one university hospital in

				Finland... The target group consisted of healthcare professionals working with potential recipients of PC… Data collection took place between November and December 2024…
Participants	6	(*a*) *Cohort study*—Give the eligibility criteria, and the sources and methods of selection of participants. Describe methods of follow-up<br>*Case-control study*—Give the eligibility criteria, and the sources and methods of case ascertainment and control selection. Give the rationale for the choice of cases and controls<br>*Cross-sectional study*—Give the eligibility criteria, and the sources and methods of selection of participants	4	The inclusion criteria were a profession and a ward or unit in which the PC competence described by THL may be relevant…
		(*b*) *Cohort study*—For matched studies, give matching criteria and number of exposed and unexposed<br>*Case-control study*—For matched studies, give matching criteria and the number of controls per case	N/A	
Variables	7	Clearly define all outcomes, exposures, predictors, potential confounders, and effect modifiers. Give diagnostic criteria, if applicable	4–6	The questionnaire contained background questions (n=13) on ward or unit, profession… categorical variables (profession; ward or unit; work experience in healthcare as a whole…
Data sources/measurement	8*	For each variable of interest, give sources of data and details of methods of assessment (measurement). Describe comparability of assessment methods if there is more than one group	6	Descriptive analyses included frequencies and percentages for categorical variables… comparisons between groups…
Bias	9	Describe any efforts to address potential sources of bias	4	To obtain responses from as wide a range of professions and wards or units as possible, the sample

				size was not predetermined.
Study size	10	Explain how the study size was arrived at	4	To obtain responses from as wide a range of professions and wards or units as possible, the sample size was not predetermined.

Quantitative variables	11	Explain how quantitative variables were handled in the analyses. If applicable, describe which groupings were chosen and why	5–6	For the analysis, participants were grouped by profession… Descriptive analyses included frequencies… comparisons between groups…
Statistical methods	12	(*a*) Describe all statistical methods, including those used to control for confounding	5–6	Initial construct validity testing was performed using exploratory factor analysis… The data analysis was conducted…
		(*b*) Describe any methods used to examine subgroups and interactions	N/A	
		(*c*) Explain how missing data were addressed	Tables 1 & 5, p. 13	Items 3.3–3.7 have been excluded from the sum variables due to only applying to certain participants based on profession… Since the electronic survey tool used prevents participants from returning an incomplete form, there were no missing responses in the data.
		(*d*) *Cohort study*—If applicable, explain how loss to follow-up was addressed<br>*Case-control study*—If applicable, explain how matching of cases and controls was addressed<br>*Cross-sectional study*—If applicable, describe analytical methods taking account of sampling strategy	N/A	

		(*e*) Describe any sensitivity analyses	N/A	
**Results**				
Participants	13*	(a) Report numbers of individuals at each stage of study—eg numbers potentially eligible, examined for eligibility, confirmed eligible, included in the study, completing follow-up, and analysed	7, 14	A total of 100 participants responded to the questionnaire, 62% of whom were nurses… Out of 5,000 healthcare professionals fulfilling the inclusion criteria…
		(b) Give reasons for non-participation at each stage	14–15	…the questionnaire was in Finnish only… since the invitation was distributed via several contact persons using different mailing lists, ascertaining the exact number of potential respondents reached proved impossible… In addition, online surveys generally have low response rates…
		(c) Consider use of a flow diagram	N/A	
Descriptive data	14*	(a) Give characteristics of study participants (eg demographic, clinical, social) and information on exposures and potential confounders	7	A total of 100 participants responded to the questionnaire, 62% of whom were nurses…
		(b) Indicate number of participants with missing data for each variable of interest	Tables 1 & 5	Items 3.3–3.7 excluded due to only being directed at certain respondents.
		(c) *Cohort study*—Summarise follow-up time (eg, average and total amount)	N/A	
Outcome data	15*	*Cohort study*—Report numbers of outcome events or summary measures over time	N/A	
		Case-control study—Report numbers in each exposure category, or summary measures of exposure	N/A	
		Cross-sectional study—Report numbers of outcome events or summary measures	7	A total of 100 participants responded to the questionnaire, 62% of whom were nurses…
Main results	1	(*a*) Give unadjusted estimates and, if applicable, confounder-	7–8	A total of 100

	16	adjusted estimates and their precision (eg, 95% confidence interval). Make clear which confounders were adjusted for and why they were included		participants responded to the questionnaire, 62% of whom were nurses…
		(*b*) Report category boundaries when continuous variables were categorized	6	Work experience, both in healthcare overall and in PC specifically, was stratified into three groups…
		(*c*) If relevant, consider translating estimates of relative risk into absolute risk for a meaningful time period	N/A	
Other analyses	17	Report other analyses done—eg analyses of subgroups and interactions, and sensitivity analyses	N/A	
**Discussion**				
Key results	18	Summarise key results with reference to study objectives	11–12	Most of the items (17 out of 30) were rated "Good" among all respondents…
Limitations	19	Discuss limitations of the study, taking into account sources of potential bias or imprecision. Discuss both direction and magnitude of any potential bias	13–15	The study has some limitations as well. The inclusion of several professional groups may also be seen as a weakness…
Interpretation	20	Give a cautious overall interpretation of results considering objectives, limitations, multiplicity of analyses, results from similar studies, and other relevant evidence	10–16	Although previous studies have examined PC competence … The results suggest that healthcare professionals feel the need for more education on some aspects of palliative and end-of-life care…
Generalisability	21	Discuss the generalisability (external validity) of the study results	13–15	The instrument was based on Finnish guidelines…
**Other information**				
Funding	22	Give the source of funding and the role of the funders for the present study and, if applicable, for the original study on which the present article is based	Title Page	This study did not receive any external funding.

*Give information separately for cases and controls in case-control studies and, if applicable, for exposed and unexposed groups in cohort and cross-sectional studies.

Note: An Explanation and Elaboration article discusses each checklist item and gives methodological background and published examples of transparent reporting. The STROBE checklist is best used in conjunction with this article (freely available on the Web sites of PLoS Medicine at http://www.plosmedicine.org/, Annals of Internal Medicine at http://www.annals.org/, and Epidemiology at http://www.epidem.com/). Information on the STROBE Initiative is available at www.strobe-statement.org.
