# Supplementary material for: Self-assessed palliative care competence of healthcare professionals in a Finnish university hospital: A mixed methods study
Source: Palliat Care Soc Pract. 2026 Aug 25;20:26323524261480156. doi: 10.1177/26323524261480156 (PMC13519223; doi:10.1177/26323524261480156)
Supplement: Supplementary file 3 [file sj-pdf-3-pcr-10.1177_26323524261480156.pdf]

Supplementary file 1. Questionnaire used in the survey.

Finnish (original)

Tutkittavan suostumus tutkimukseen

Palliatiivisen hoidon osaaminen, kysely terveydenhuollon henkilöstölle

Hyvä vastaaja,

Sosiaali- ja terveysministeriön rahoittaman [HANKKEEN NIMI] yhteydessä kartoitetaan palliatiivista hoitoa ja saattohoitoa toteuttavien terveydenhuollon ammattilaisten palliatiivisen hoidon ja saattohoidon osaamista sekä koulutustarpeita.

Kyselyssä osaamista tarkastellaan perustuen THL:n (2022) Palliatiivisen hoidon ja saattohoidon kansallinen laatusuositus -julkaisussa kuvattuun suositukseen hyvän palliatiivisen hoidon ja saattohoidon laatukriteereiksi. Kyselyssä käytetty jaottelu seitsemään laatualueeseen vastaa laatusuosituksen rinnalla ilmestyneessä Laatukäsikirja elämän loppuvaiheen hoitoon iäkkäiden ihmisten palveluissa – julkaisussa (THL, 2022) kuvattuja laatualueita.

Kyselyyn vastaaminen on vapaaehtoista. Tutkimuksessa ei kerätä sinulta henkilötietoja ja tutkimuksessa kerättävät aineistot ovat ainoastaan tutkimuksen tutkimusryhmän tiedossa. Tutkimusryhmän jäsenet ovat salassapitovelvollisia. Kaikkia tutkimustuloksia käsitellään siten, ettei yksittäisiä vastaajia pystytä tunnistamaan tuloksista. Tutkimusaineistosta tallennetaan vain välttämättömiä tietoja ja aineisto säilytetään salasanoin suojatussa palvelimessa. Kerättyä tietoa käytetään ainoastaan tämän tutkimuksen toteuttamiseen. Tutkimuksen tuloksia käytetään [SAIRAALAN NIMI] palliatiivisen hoidon osaamisen kehittämiseen ja palliatiivisen keskuksen suunnittelun tueksi. Lisäksi tuloksista voidaan kirjoittaa julkaisuja ja tuloksia voidaan esitellä esimerkiksi seminaareissa. Pyydämme Sinua ystävällisesti vastaamaan kyselyyn. Vastaamiseen kuluu aikaa n. 10–15 minuuttia.

Kyselyyn voi vastata 9.12.2024 asti. Kaikki vastaukset käsitellään anonyymisti.

Kiitos jo etukäteen! Lisätietoja [KIRJOITTAJA 1]

[KIRJOITTAJA 2]

TIETOINEN SUOSTUMUS

Olen perehtynyt tutkimuksen tiedotteeseen ja annan tietoisen suostumuksen osallistumisesta tutkimukseen.

◊ Kyllä

Nykyinen pääasiallinen työyksikköni

◊ [SAIRAALAN NIMI] Aistinelinsairauksien keskus
◊ [SAIRAALAN NIMI] Lasten ja naisten osaamiskeskus
◊ [SAIRAALAN NIMI] Geriatrian ja yleislääketieteen osaamiskeskus
◊ [SAIRAALAN NIMI] Leikkaus- ja anestesiakeskus
◊ [SAIRAALAN NIMI] Tehohoitokeskus
◊ [SAIRAALAN NIMI] Sydänkeskus
◊ [SAIRAALAN NIMI] Neurokeskus
◊ [SAIRAALAN NIMI] Sisätautikeskus
◊ [SAIRAALAN NIMI] Syöpähoitokeskus
◊ [SAIRAALAN NIMI] Päivystyskeskus
◊ [SAIRAALAN NIMI] Tukielinkirurgian keskus
◊ [SAIRAALAN NIMI] Vatsakeskus
◊ [SAIRAALAN NIMI] Ensihoito
◊ Muu, mikä?

Nykyinen työtehtäväni

◊ Lähihoitaja tai perushoitaja
◊ Sairaanhoitaja
◊ Terveydenhoitaja
◊ Kätilö
◊ Ensihoitaja
◊ Fysioterapeutti
◊ Toimintaterapeutti
◊ Kuntoutusohjaaja
◊ Lääkäri
◊ Muu, mikä?

Koulutus (valitse kaikki koulutukset, jotka olet suorittanut)

◊ Kouluasteen/nuorisoasteen ammatillinen tutkinto (esim. lähihoitaja, perushoitaja)
◊ Opistoasteen tutkinto/ammattikorkeakoulututkinto (amk)
◊ Ammattikorkeakoulun jatkotutkinto (ylempi amk)
◊ Yliopiston alempi korkeakoulututkinto (esim. kandidaatti)
◊ Yliopiston ylempi korkeakoulututkinto (esim. maisteri, lisensiaatti)
◊ Yliopiston tohtorintutkinto
◊ Muu, mikä?

Hoidan työssäni palliatiivista hoitoa tai saattohoitoa saavia potilaita (valitse yksi parhaiten sopiva):

- ◊ Päivittäin
- ◊ Viikoittain
- ◊ Kuukausittain
- ◊ Muutaman kerran vuodessa
- ◊ En koskaan

Olen saanut palliatiiviseen hoitoon ja saattohoitoon liittyvää täydennyskoulutusta (voit valita useamman vaihtoehdon):

- ◊ En koskaan
- ◊ En riittävästi
- ◊ Riittävästi
- ◊ Saan säännöllisesti koulutusta aiheesta

Työkokemus sosiaali- ja terveydenhuollossa kokonaisuudessaan (valitse yksi):

- ◊ ei työkokemusta
- ◊ alle 2 vuotta
- ◊ 2–5 vuotta
- ◊ 6–10 vuotta
- ◊ 11–15 vuotta
- ◊ yli 15 vuotta
-

Työkokemus palliatiivisesta hoidosta ja/tai saattohoidosta (valitse yksi):

- ◊ ei työkokemusta
- ◊ alle 2 vuotta
- ◊ 2–5 vuotta
- ◊ 6–10 vuotta
- ◊ 11–15 vuotta
- ◊ yli 15 vuotta

Olen suorittanut THL:n saattohoitopassin (valitse yksi):

- ◊ Kyllä
- ◊ En

Olen osana perehdytystä saanut tietoa palliatiivisessa hoidossa olevan henkilön oireiden hoidosta (valitse yksi):

- Kyllä
- En
- En osaa sanoa

Tiedän organisaationi palliatiivista hoitoa ja saattohoitoa koskevat konsultaatiokäytännöt (valitse yksi):

- Kyllä
- En
- En osaa sanoa

Osaan tarvittaessa konsultoida eri tahoja palliatiivista hoitoa saavan tai saattohoidossa olevan potilaan hoitoon liittyvissä asioissa (valitse yksi):

- Kyllä
- En
- En osaa sanoa

Saan tarpeen mukaan työnohjausta palliatiivisen hoitoon tai saattohoitoon liittyen (valitse yksi):

- Kyllä
- En
- En osaa sanoa

Tarvitsen työssäni palliatiivisen hoidon ja saattohoidon osaamista:

- Täysin eri mieltä
- Osittain eri mieltä
- En samaa enkä eri mieltä
- Osittain samaa mieltä
- Täysin samaa mieltä
- En osaa sanoa

**Laatualue 1. Hoitotahto ja asiakaskeskeinen työ**<br><br>Osaan…	Osaan erinomaisesti	Osaan kiitettävästi	Osaan hyvin	Osaan kohtalaisesti	Osaan heikosti	Ei koske minun työnkuvaani
…ohjata potilasta hoitotahtoon liittyvissä asioissa.						
…huomioida hoitolinjaukset potilaan hoidossa tehtävänkuvani mukaisesti.						
…huomioida hoidonrajaukset potilaan hoidossa tehtävänkuvani mukaisesti.						
…antaa potilaalle riittävästi tietoa hänen lääketieteellisestä tilanteestaan tehtävänkuvani mukaisesti.						

**Laatualue 2.**	Osaan	Osaan	Osaan	Osaan	Osaan	Ei koske

**Läheisten rooli ja vuorovaikutus henkilöstön kanssa**<br><br>Osaan…	erinomaisesti	kiitettävästi	hyvin	kohtalaisesti	heikosti	minun työnkuvaani
…tukea palliatiivista hoitoa saavan tai saattohoidossa olevan potilaan läheisiä heidän yksilöllisissä tarpeissaan.						
… ohjata palliatiivista hoitoa saavan tai saattohoidossa olevan potilaan läheisiä.						
… antaa palliatiivista hoitoa saavan tai saattohoidossa olevan potilaan läheisille tarvittavaa tietoa.						
…huomioida edunvalvonnan roolin ja merkityksen palliatiiviseen hoitoon liittyvässä päätöksenteossa.						

**Laatualue 3. Elämän loppuvaiheen ennakoiva hoitosuunnitelma ja hoitolupaus**<br><br>Osaan…	Osaan erinomaisesti	Osaan kiitettävästi	Osaan hyvin	Osaan kohtalaisesti	Osaan heikosti	Ei koske minun työnkuvaani
…sanoa, mitä elämän loppuvaiheen ennakoivan hoitosuunnitelman tulee sisältää.						
…huomioida elämän loppuvaiheen ennakoivan hoitosuunnitelman						

toteuttaessani hoitoa.						
HOITAJAT:<br>- …arvioida elämän loppuvaiheen ennakoivan hoitosuunnitelman päivittämistarpeet.<br>- …tarvittaessa käynnistää elämän loppuvaiheen ennakoivan hoitosuunnitelman päivittämisprosessin.<br><br>LÄÄKÄRIT:<br>- …arvioida, milloin elämän loppuvaiheen ennakoivan hoitosuunnitelman laatiminen on tarpeellista.<br>- …laatia elämän loppuvaiheen ennakoivan hoitosuunnitelman.<br>- …laatia lääketieteelliset hoidonrajaukset sekä antaa ennakoivat hoito-ohjeet.						

**Laatualue 4. Osaamisen turvaaminen**<br><br>Osaan…	Osaan erinomaisesti	Osaan kiitettävästi	Osaan hyvin	Osaan kohtalaisesti	Osaan heikosti	Ei koske minun työnkuvaani
…tunnistaa palliatiivista hoitoa saavan tai saattohoidossa olevan potilaan oireita.						
…hoitaa palliatiivista						

hoitoa saavan tai saattohoidossa olevan potilaan oireita lääkkeettömin keinoin.						
…hoitaa palliatiivista hoitoa saavan tai saattohoidossa olevan potilaan oireita lääkkeellisin keinoin.						
…tunnistaa lähestyvän kuoleman merkkejä.						

**Laatualue 5. Asiantuntijuuden saatavuus ja hoitoketjun varmistaminen**<br><br>Osaan…	Osaan erinomaisesti	Osaan kiitettävästi	Osaan hyvin	Osaan kohtalaisesti	Osaan heikosti	Ei koske minun työnkuvaani
…tunnistaa palliatiivista hoitoa saavan tai saattohoidossa olevan potilaan psykososiaaliset tarpeet.						
…tarvittaessa järjestää palliatiivista hoitoa saavalle tai saattohoidossa olevalle potilaalle psykososiaalisiin tarpeisiin liittyvää asiantuntijan apua.						
…tunnistaa palliatiivista hoitoa saavan tai						

saattohoidossa olevan potilaan henkiset/hengelliset tarpeet.						
…tarvittaessa järjestää palliatiivista hoitoa saavalle tai saattohoidossa olevalle potilaalle henkisiin/hengellisiin tarpeisiin liittyvää asiantuntijan apua.						

**Laatualue 6. Rakenteelliset muut tekijät ja apuvälineet**<br><br>Osaan…	Osaan erinomaisesti	Osaan kiitettävästi	Osaan hyvin	Osaan kohtalaisesti	Osaan heikosti	Ei koske minun työnkuvaani
…käyttää hoito- ja apuvälineitä palliatiivisessa hoidossa ja saattohoidossa.						
…tunnistaa potilaan yksilölliset hoidon tarpeet ja toiveet esimerkiksi yksityisyyden suhteen.						
…osallistaa läheisiä palliatiivista hoitoa saavan tai saattohoidossa olevan potilaan hoitoon potilaan niin halutessa.						

**Laatualue 7. Kuoleman tapahtuessa ja tapahduttua**<br><br>Osaan…	Osaan erinomaisesti	Osaan kiitettävästi	Osaan hyvin	Osaan kohtalaisesti	Osaan heikosti	Ei koske minun työnkuvaani

…toimia potilaan kuoleman tapahtuessa.						
…lohduttaa ja tukea läheisensä menettäneitä.						
…ohjata läheisiä kuoleman jälkeisten käytännön asioiden hoitamisessa (esim. asiointi hautaustoimiston kanssa).						
…ohjata kuoleman kohdanneita läheisiä tuen piiriin (esim. keskusteluapu, vertaistuki).						

Koen tarvitsevani lisää koulutusta seuraavista sisällöistä (voit valita useamman vaihtoehdon):

- **Hoitotahto ja asiakaskeskeinen työ**
  - Hoitotahto
  - Hoidon linjaukset
  - Hoidonrajaukset
  - Tiedon antaminen potilaan lääketieteellisestä tilasta
- **Läheisten rooli ja vuorovaikutus henkilöstön kanssa**
  - Läheisten tukeminen
  - Tiedon antaminen läheisille
  - Edunvalvonnan rooli ja merkitys
- **Elämän loppuvaiheen ennakoiva hoitosuunnitelma ja hoitolupaus**
  - Elämän loppuvaiheen ennakoivan hoitosuunnitelman sisältö ja huomioiminen hoidon toteuttamisessa
  - Elämän loppuvaiheen ennakoivan hoitosuunnitelman päivittämistarpeet
  - Elämän loppuvaiheen ennakoivan hoitosuunnitelman ja hoidonrajausten laatiminen
- **Osaamisen turvaaminen**
  - Oireiden tunnistaminen
  - Oireiden lääkkeetön ja lääkkeellinen hoitaminen
  - Lähestyvän kuoleman merkkien tunnistaminen
- **Asiantuntijuuden saatavuus ja hoitoketjun varmistaminen**
  - Psykososiaalisten ja henkisten/hengellisten tarpeiden tunnistaminen
  - Asiantuntijan avun järjestäminen psykososiaalisen tai henkisen/hengellisen avun tarpeeseen
- **Rakenteelliset muut tekijät ja apuvälineet**

    - Hoito- ja apuvälineiden käyttäminen
    - Yksilölliset tarpeet ja toiveet esimerkiksi yksityisyyden suhteen
    - Läheisten osallistaminen
- **Kuoleman tapahtuessa ja tapahduttua**
    - Toimiminen kuoleman tapahtuessa
    - Läheisensä menettäneiden tukeminen
    - Läheisten ohjaaminen kuoleman jälkeisiin toimenpiteisiin ja tukeen liittyen
- **Jokin muu, mikä? ___________________________________**
- **Ei mikään näistä**

Mitä muuta haluan kertoa palliatiivisen hoidon osaamisestani?

Palautteeni tästä kyselystä:

English (unofficial translation by the author)

Consent to participate in the study

Palliative care competence, survey for healthcare professionals

Dear respondent,

Alongside the [NAME OF PROJECT] funded by the Ministry of Social Affairs and Health, we are charting the palliative and end-of-life care competence and educational needs of healthcare professionals providing palliative and end-of-life care.

In the survey, competence is examined based on the recommendations regarding the quality criteria for good palliative and end-of-life care as described in the publication " National quality recommendation for palliative care and end of life care" by The Finnish Institute for Health and Welfare (THL) (2022). The division into seven Quality Areas used in the questionnaire corresponds to the Quality Areas described in the publication "The quality handbook for end-of-life care in services for older people" (THL, 2022) published alongside the quality recommendation.

Responding to the survey is voluntary. No personal data will be colleted from you for the survey, and the data collected are available to the research team only. All members of the research team are bound by confidentiality. All research results will be processed so that no individual respondents can be identified from the results. Nothing but strictly necessary data will be stored, and the data will be stored on a password-protected server. The data collected will only be used for the purposes of this study. The study results will be used to improve palliative care competence at [NAME OF HOSPITAL] and as support for developing the palliative centre. Additionally, the results may be written about in publications, and the results may be presented e.g. in seminars. We kindly ask you to respond to the questionnaire. Responding will take about 10–15 minutes.

The questionnaire is open until 9th Dec, 2024. All responses will be processed anonymously.

Thank you in advance! More information: [AUTHOR 1]

[AUTHOR 2]

INFORMED CONSENT

I have been acquainted with the participant information sheet and give my informed consent to participate in the study.

- ◊ Yes

My current primary work unit

- ◊ [NAME OF HOSPITAL] Sensory Organ Diseases
- ◊ [NAME OF HOSPITAL] Gynaeocology and Pediatrics
- ◊ [NAME OF HOSPITAL] Geriatrics and General Medicine
- ◊ [NAME OF HOSPITAL] Surgery and Anaesthesia
- ◊ [NAME OF HOSPITAL] Intensive Care
- ◊ [NAME OF HOSPITAL] Cardiology
- ◊ [NAME OF HOSPITAL] Neurology
- ◊ [NAME OF HOSPITAL] Internal Medicine
- ◊ [NAME OF HOSPITAL] Oncology
- ◊ [NAME OF HOSPITAL] Emergency Department
- ◊ [NAME OF HOSPITAL] Musculoskeletal Surgery
- ◊ [NAME OF HOSPITAL] Gastrology
- ◊ [NAME OF HOSPITAL] Prehospital Emergency Medical Care
- ◊ Other, please specify:

My current professional role

- ◊ Practical nurse
- ◊ Registered nurse
- ◊ Public health nurse
- ◊ Midwife
- ◊ Emergency Medical Technician
- ◊ Physical therapist
- ◊ Occupational therapist
- ◊ Rehabilitation specialist
- ◊ Physician
- ◊ Other, please specify:

Education (select all you have completed)

- ◊ Vocational degree (e.g. practical nurse)
- ◊ University of Applied Sciences degree, lower (Bachelor’s degree)
- ◊ University of Applied Sciences degree, higher (Master’s degree)
- ◊ University-level degree, lower (e.g. Bachelor’s degree)
- ◊ University-level degree, higher (e.g. Master’s degree, Licentiate of Medicine)
- ◊ University-level doctoral degree
- ◊ Other, please specify:

In my work, I care provide care for patients receiving palliative or end-of-life care (select the most applicable one):

◊ Daily
◊ Weekly
◊ Monthly
◊ A few times per year
◊ Never

I have received supplementary training regarding palliative and end-of-life care (you may select more than one):

◊ Never
◊ Not sufficiently
◊ Sufficiently
◊ I regularly receive training on the topic

Work experience in healthcare and social welfare overall (please select one):

◊ no work experience
◊ less than 2 years
◊ 2–5 years
◊ 6–10 years
◊ 11–15 years
◊ more than 15 years

Work experience in palliative and/or end-of-life care (please select one):

◊ no work experience
◊ less than 2 years
◊ 2–5 years
◊ 6–10 years
◊ 11–15 years
◊ more than 15 years

I have completed the THL *Saattohoitopassi* course (please select one):

◊ Yes
◊ No

During my orientation, I have received information about treating the symptoms of those receiving palliative care (please select one):

◊ Yes
◊ No
◊ Unsure

I am familiar with the consulation practices of my organisation concerning palliative and end-of-life care (please select one):

◊ Yes
◊ No
◊ Unsure

I am able to consult various parties on matters concerning patients receiving palliative or end-of-life care when necessary (please select one):

- ◊ Yes
- ◊ No
- ◊ Unsure

I can receive clinical supervision regarding palliative and end-of-life care when necessary (please select one):

- ◊ Yes
- ◊ No
- ◊ Unsure

I need palliative and end-of-life care competence in my work:

- ◊ Completely disagree
- ◊ Partly disagree
- ◊ Neither agree nor disagree
- ◊ Partly agree
- ◊ Completely agree
- ◊ Unsure

**Quality area 1. Living will and patient-centred work**<br><br>I am able to…	Excellent	Very good	Good	Fair	Poor	Not applicable to my job description
…guide the patient on matters pertaining to living will.						
… Take treatment intent into consideration when providing care, as required by my job description.						
… Take treatment limitations [e.g. do-not-resuscitate (DNR) orders] into consideration when providing care, as required by my job description.						

… Provide the patient with information regarding their medical state, as required by my job description.						

**Quality area 2. Role of the patient's next of kin and their interaction with the personnel**<br><br>I am able to…	Excellent	Very good	Good	Fair	Poor	Not applicable to my job description
… Support the next of kin of a patient receiving palliative or end-of-life care regarding their individual needs.						
… Provide guidance to the next of kin of a patient receiving palliative or end-of-life care.						
… Provide the next of kin of a patient receiving palliative or end-of-life care with pertinent information.						
… Take the role and significance of guardianship into consideration in decision-making related to palliative care.						

**Quality area 3. Advance care plan and promise of care continuity**	Excellent	Very good	Good	Fair	Poor	Not applicable to my job description

I am able to…						
… Describe what an advance care plan should contain.						
… take the patient’s advance care plan into account when providing care.						
NURSES:<br>- … Recognise the need to update the patient’s advance care plan.<br>- … Start the process of updating the patient’s advance care plan if needed.<br><br>PHYSICIANS:<br>- … Recognise the need for an advance care plan.<br>- Draw up an advance care plan.<br>- … Formulate medical treatment limitations and provide anticipatory care instructions.						

**Quality area 4. Ensuring competence**<br><br>I am able to…	Excellent	Very good	Good	Fair	Poor	Not applicable to my job description
…Recognise symptoms exhibited by a patient receiving palliative or end-of-life care.						
…Treat symptoms exhibited by a patient receiving palliative or end-of-life care by nonpharmacological means.						

… Treat symptoms exhibited by a patient receiving palliative or end-of-life care by pharmacological means.						
…Recognise the signs of approaching death.						

**Quality area 5. Availability of experts and securing the service chain**<br><br>I am able to…	Excellent	Very good	Good	Fair	Poor	Not applicable to my job description
…Recognise the psychosocial needs of a patient receiving palliative or end-of-life care.						
… Provide a patient receiving palliative or end-of-life care with expert support for psychosocial needs.						
…Recognise the existential/spiritual needs of a patient receiving palliative or end-of-life care.						
… Provide a patient receiving palliative or end-of-life care with expert support for existential/spiritual needs.						

**Quality area 6. Other structural factors and assistive devices**<br><br>I am able to…	Excellent	Very good	Good	Fair	Poor	Not applicable to my job description
… Use assistive devices and equipment in palliative and end-of-life care.						
…Recognise the patient's individual needs and requests regarding matters such as privacy.						
… Give the next of kin of a patient receiving palliative or end-of-life care the opportunity to participate in care, if the patient so wishes.						

**Quality area 7. In the event of and after death**<br><br>I am able to…	Excellent	Very good	Good	Fair	Poor	Not applicable to my job description
…Act in the event of death.						
…Support and console the bereaved.						
…Guide the patient's next of kin on practical matters following the patient's passing, e.g. issues related to funeral arrangements.						
…Direct the bereaved to appropriate sources of support.						

I feel I need more education on the following topics (you may select more than one):

- ◊ **Living will and patient-centred work**
  - o Living will
  - o Treatment intent
  - o Treatment limitations
  - o Providing information regarding the patient's medical state
- ◊ **Role of the patient's next of kin and their interaction with the personnel**
  - o Providing support to the patient's next of kin
  - o Providing information to the patient's next of kin
  - o Role and significance of guardianship
- ◊ **Advance care plan and promise of care continuity**
  - o Content of advance care plan and taking the plan into account while providing care
  - o Need to update the advance care plan
  - o Making an advance care plan and setting treatment limitations
- ◊ **Ensuring competence**
  - o Recognising symptoms
  - o Treating symptoms using nonpharmacological and pharmacological means
  - o Recognising the signs of approaching death
- ◊ **Availability of experts and securing the service chain**
  - o Recognising psychosocial and existential/spiritual needs
  - o Arranging for expert support for those in need of psychosocial and existential/spiritual support
- **Other structural factors and assistive devices**
  - o Using assistive and treatment devices
  - o Individual needs and requests e.g. regarding privacy
  - o Enabling the participation of the patient's next of kin
- **In the event of and after death**
  - o Acting in the event of death
  - o Providing support to the bereaved
  - o Poviding guidance to the bereaved regarding procedures and support following he patient's passing
- **Other, please specify: ____________________________________**
- **None of the above**

What else would I like to share about my palliative care competence?

My feedback regarding this questionnaire:

